# Global aspirations, local realities: the role of social science research in controlling neglected tropical diseases

**DOI:** 10.1186/2049-9957-3-35

**Published:** 2014-10-01

**Authors:** Kevin Bardosh

**Affiliations:** Centre of African Studies, School of Social and Political Science, College of Humanities and Social Science, The University of Edinburgh, 58 George Square, Edinburgh, EH8 9LD UK; Division of Pathway Medicine and Centre for Infectious Diseases, School of Biomedical Sciences, College of Medicine and Veterinary Medicine, The University of Edinburgh, Chancellor’s Building, 49 Little France Crescent, Edinburgh, EH16 4SB UK

**Keywords:** Neglected tropical diseases, Applied social sciences, Implementation research, Social determinants, Community participation, Policy, Global health, Sociology, Anthropology

## Abstract

Neglected Tropical Diseases (NTDs) are both drivers and manifestations of poverty and social inequality. Increased advocacy efforts since the mid-2000s have led to ambitious new control and elimination targets set for 2020 by the World Health Organisation. While these global aspirations represent significant policy momentum, there are multifaceted challenges in controlling infectious diseases in resource-poor local contexts that need to be acknowledged, understood and engaged. However a number of recent publications have emphasised the “neglected” status of applied social science research on NTDs. In light of the 2020 targets, this paper explores the social science/NTD literature and unpacks some of the ways in which social inquiry can help support effective and sustainable interventions. Five priority areas are discussed, including on policy processes, health systems capacity, compliance and resistance to interventions, education and behaviour change, and community participation. The paper shows that despite the multifaceted value of having anthropological and sociological perspectives integrated into NTD programmes, contemporary efforts underutilise this potential. This is reflective of the dominance of top-down information flows and technocratic approaches in global health. To counter this tendency, social research needs to be more than an afterthought; integrating social inquiry into the planning, monitoring and evaluating process will help ensure that flexibility and adaptability to local realities are built into interventions. More emphasis on social science perspectives can also help link NTD control to broader social determinants of health, especially important given the major social and economic inequalities that continue to underpin transmission in endemic countries.

**Electronic supplementary material:**

The online version of this article (doi:10.1186/2049-9957-3-35) contains supplementary material, which is available to authorized users.

## Multilingual abstracts

Please see Additional file 1 for translations of the abstract into the six official working languages of the United Nations.

## Background

Influenced by certain ecological conditions and transmitted through various vectors and animals, Neglected Tropical Diseases (NTDs) are a heterogeneous group of some 17 parasitic, bacterial, viral and fungal infections that burden the poor and marginalised in developing countries where they cause much human suffering and poverty. One billion people, referred to as the “bottom billion”, are estimated to be infected by the seven most prevalent NTDs (schistosomiasis, trachoma, soil-transmitted helminths, lymphatic filariasis and onchocerciasis) in over 100 countries [1–3]. Taken together, NTDs are thought to be second to HIV/AIDS in terms of infectious disease burden yet they receive only a small proportion of development assistance allocated to health [4]. The impact of these infectious pathogens is often difficult to quantify due to under-reporting, focal clustering, poly-parasitism, diverse morbidity, stigmatisation and multifaceted influences on local livelihoods. Although notable gaps in control and treatment tools remain, many NTDs have a practical “toolbox” of control options; this ranges from mass anthelmintic treatment of populations, vector control, active case detection and treatment, targeting of the animal reservoir, environmental modification and improvements in housing, water, hygiene and sanitation [2].

Since the early-2000s, advocacy efforts have increased greatly for NTDs, and these “forgotten afflictions” have become somewhat less neglected in the global health landscape [3]. Much of this new attention has been driven by policy narratives that have framed NTD control as an avenue towards addressing global inequity and poverty through cost-effective but big impact interventions – what have been termed “low-hanging fruit”. Such advocacy has relied heavily on the “value for money” of the integrated control of parasitic worms through mass drug administration (MDA) [5, 6]. NTD control has been linked to meeting multiple Millennium Development Goals (MDGs); numerous public-private partnerships, greater funding into scientific research and drug/diagnostic product development, significant donation of medicines by the pharmaceutical industry, and new and reinvigorated donor and country programmes have followed. Illustrative of this global momentum, the World Health Organisation (WHO) Director-General, Dr Margaret Chan, has called NTD efforts a “Cinderella tale…of moving from rags-to-riches” [7]. Both the London Declaration in 2012 and a landmark WHO resolution in 2013 signified the political and financial commitment of endemic country governments and key donors (i.e. USAID, Gates Foundation and DFID) to support ambitious new targets for control and elimination set for 2020 [8, 9]; these are summarised in Table 1 for all 17 major NTDs.

**Table 1:** **Major NTD control targets set for 2020 by WHO**

NTD	WHO Target[1]
Dengue	“With new tools for diagnosis and vector control, better case management and focused research…an integrated vector management approach should reduce rates of morbidity by at least 25% and of mortality by 50% by 2020.”
Rabies	“Elimination of human rabies transmitted by dogs and dog-to-dog transmission is achievable by 2015 in all endemic areas in Latin America; and by 2020 in all affected countries in WHO’s South-East Asia and Western Pacific regions.”
Trachoma	“…global elimination goal by 2020…By 2020, all countries will have achieved the UIG and be free from blinding trachoma as a public-health problem, and by 2020, 75% of countries will have been verified as free from blinding trachoma as a public-health problem.”
Buruli ulcer	“WHO aims to cure 70% of all cases with antibiotics in all endemic countries by 2020.”
Endemic treponematoses	“Elimination of yaws in Africa is feasible by 2020, therefore leading to global eradication”
Leprosy	“Vigorous case finding and treatment would lead to global interruption of transmission by 2020.”
Chagas disease	“A milestone will be reached when peri-domiciliary infestation has been eliminated in Latin America by 2020.”
Human African Trypanosomiasis	“…eliminate the disease in 80% of foci by 2015 and achieve elimination in 100% of foci by 2020.”
Leishmaniasis	“WHO aims to detect at least 70% of all cases of cutaneous leishmaniasis and treat at least 90% of all detected cases in the Eastern Mediterranean Region by 2015. With sustained efforts on the Indian sub-continent, 100% case-detection and treatment of visceral leishmaniasis is feasible by 2020…”
Cysticercosis	“A validated strategy for the control and elimination of *Taenia solium* taeniasis/cysticercosis will be available by 2015; and interventions for control and elimination scaled up in selected countries in Africa, Asia and Latin America by 2020.”
Dracunculiasis	“Dracunculiasis is now on the verge of eradication.”
Echinococcosis	“Pilot projects to validate the effectiveness of echinococcosis/hydatidosis control strategies will be implemented in selected countries by 2015. Scale up of interventions in selected countries in Central Asia, North Africa and Latin America for control and elimination as a public-health problem will be in place by 2020.”
Foodborne trematode infections	“By 2020, 75% of the at-risk population will have been reached by preventive chemotherapy and morbidity associated with foodborne trematode infections will be under control in 100% of the endemic countries.”
Lymphatic filariasis	“By 2020, 100% of all endemic countries will have been verified as free of transmission or will have entered post-intervention surveillance.”
Onchocerciasis	“It is currently estimated that, by 2020, 12 APOC countries and 11 ex-OCP countries may have achieved elimination, out of a total of 31 countries affected…”
Schistosomiasis	“…could be eliminated as a public health problem in multiple countries in Africa by 2020, and globally by 2025.”
Soil-transmitted helminthiases	“…75% coverage will be reached in all countries by 2020.”

Despite this enthusiasm, numerous challenges to meeting these new targets exist. These involve both technical questions about the efficacy of existing tools as well as operational issues in moving global health interventions from international boardrooms into socio-economically and politically marginalised communities. Invoking the classic dichotomisation between “top-down” and “bottom-up” public health approaches, Spiegel and others [10] have argued that current NTD programmes over-prioritise narrow technical solutions at the expense of prevention efforts and broader engagement with the social determinants of health. Debates have emerged most visibly in the rapid scaling-up of integrated MDA, which treated a reported 887.8 million people in 2009 [3, 11]. Some have questioned the scientific evidence-base of the MDA strategy [12], potential development of drug resistance [13], the feasibility of integrating several existing programmes [14], the effectiveness of using unpaid volunteers [15], negative effects on health systems [16], and local resistance and non-compliance with free treatments [17–21]. In the absence of integrating MDA with other strategies (i.e. water, hygiene and sanitation improvements, case management and vector control) others have argued that underlying causes are left unaddressed, perpetuating a “drug dependence” on preventative chemotherapeutic treatments [22–24]. The WHO itself has acknowledged a number of complex risks that threaten the feasibility of meeting the 2020 NTD targets; these range from socio-political trends, challenges in vector control, lack of local health system capacity, various scientific gaps and the limited amount of funding being allocated for implementation research [2].

Current debates reveal that despite policy narratives centered on the poverty-inducing effects of NTDs, relatively little research is being promoted, or undertaken, on how the context of poverty and marginalisation influences the effectiveness and outcome of control/elimination/eradication programmes [25]. Diseases of poverty are, by definition, both drivers and manifestations of poverty. They reflect not only individual risk factors, but larger structural inequalities in access to health services, infrastructure, food security, education, political voice and markets that drive poverty and maintain social and economic exclusion [26, 27]. Within these resource-poor settings, this context of poverty will invariably dictate how NTD interventions are implemented as well as the particular ways in which disease control technologies are adopted and used (or not used) by local health systems and target populations. In short, the same poverty-inducing factors that drive NTD transmission present various context-specific challenges to controlling them.

There is an increasing emphasis in global health to address the gap between the scientific efficacy of tools and strategies and their practical translation and impact into different local contexts – the so-called “implementation gap”. Optimising the impact of existing programmes is arguably one of the most cost-effective funding strategies that international agencies and national governments can take [28]. In the field of NTDs, the Special Programme for Research and Training in Tropical Diseases (TDR) – funded by UNICEF/UNDP/World Bank/WHO – has coordinated and funded much ground-breaking implementation research going back to the 1970s. Through training and small grants, TDR has stimulated the development of social and economic research into tropical diseases, brought social scientists into collaborative arrangements with biomedical scientists (especially on malaria, schistosomiasis and onchocerciasis) and worked to highlight otherwise overlooked areas, such as the importance of gender and community participation [29]. Acknowledging this contribution, two recent reports partially funded by TDR – *Implementation Research for the Control of Infectious Diseases of Poverty* in 2011 and *The Global Report for Research on Infectious Diseases of Poverty* in 2012 – have emphasised the need for greater emphasis on implementation research in NTD control [30, 31]. These reports were built explicitly on the conceptual integration of human, animal and ecosystem health as articulated by the “One World, One Health” movement and outlined a new trans-disciplinary vision for addressing, among other things, the social determinants of health and illness – and hence promoted the greater involvement of social scientists [32].

However there are a number of difficulties in realising this agenda. As with global health more generally, funding for NTDs is heavily concentrated on biomedical research and, to a lesser degree, pharmaceutical industry support for drug development [10, 33, 34]. Requiring multidisciplinary teams and new sorts of methodologies, part of the “neglect” of implementation research has to do with the fact that it often not considered a “serious science” [34–36]. The actual translation of such research into more effective interventions is also complex and uncertain. Research on gaps between policy, programmes and target populations regularly encounter barriers to being incorporated into particular programmes or the larger health system landscape; hence simply conducting research on the “implementation gap” does not mean operational practices will change and be improved.

One of the major dimensions to the implementation–NTD nexus relates to the level of involvement of social scientists – particularly sociologists and anthropologists – in interventions. Disjunctions between biomedicine and social research have traditionally been maintained by disciplinary (and epistemological) differences as well as working norms, systems of reward and grant funding cycles [36–39]. Despite efforts dating back to the 1970s [29, 39–41], a number of recent publications (some commissioned by TDR itself) have criticised the contemporary NTD landscape for a lack of social inquiry and consideration of local contexts in devising and implementing control programmes [17, 21, 25, 42–45]. In many ways, these publications have reiterated old debates about the limited cross-fertilisation between biomedical and social scientists. Allotey *et al.*[43] summarily stated that “research and interventions for neglected tropical diseases, largely neglect the social and ecological contextual, factors that make these diseases persist in the target populations” and that social research, when it is conducted, is largely “hand-maiden” to biologically-defined solutions and perspectives. Another recent paper claimed that the epistemic communities working on NTDs and social determinants have been largely “passing in the night” with little direct contact [44]. A bibliographical analysis of research publications emphasised that the social sciences are being “under-prioritised” and “neglected” by the contemporary NTD research and programme landscape [42]. Tensions have most vividly been shown in a series of publications, including in *The Lancet* (the prestigious UK medical journal), between anthropologists, who found community resistance to MDA in Uganda and Tanzania, and some longstanding NTD advocates based at the WHO and elsewhere [3, 11, 17, 45].

The aims of this paper are to move beyond polarising statements about the lack of social science research on NTDs; the paper attempts to stimulate critical debate about what types of studies have already been conducted and what they show us about *how* applied social inquiry can contribute towards supporting the 2020 targets. Using the NTD–specific literature, the paper explores some of the major challenges facing planners, implementers and communities in sustainably controlling NTDs and the barriers and bridges that require more consideration. Moving conceptually from the global into the local, it maps out five priority areas where applied sociology and anthropology can best contribute to an implementation research agenda, including on policy processes, health systems capacity, community responses to interventions, education and behaviour change and community participation. Methodologically, the paper does not attempt to systematically analyse the existing literature, which would have proven to be a more complex task considering the 17 NTDs. Rather it builds on recent reviews of social science research and NTDs mentioned above, including a bibliographical analysis of existing literature for chikungunya, dengue, visceral leishmaniasis, and onchocerciasis [42], NTDs and social determinants [27] and NTDs and community participation [41]. It supplemented these data sources with secondary bibliographic searches of key journals and other important publications, aiming to reference some of the most insightful and representative academic publications that engage with the challenges of NTD interventions. The paper mainly focuses on research published since 2000.

## Review

### I. Policy processes

The first priority area for social research involves policy processes, which remain poorly understood in most developing economies and involve various components; a useful framework developed by the Institute of Development Studies (IDS), University of Sussex [46], divides policy processes into discourses/narratives, social networks and politics/interests. Tied to a specified set of actors used to mobilise resources, policy narratives often frame issues in simplistic ways to reduce uncertainty and enrol support. These are based on specific underlying assumptions that create boundaries around an issue, define the limits of action and what/who is to be included and excluded.

Neglected diseases largely persist in countries with financial and human resource constraints. In a unique study, Spiegel *et al.*[47] reported that a central bottleneck in dengue control programmes related to value differences between stakeholders and the ability for institutions to adapt and learn from operational mistakes. More generally, bureaucratic norms and weaknesses in management have been noted as one of the major binding constraints in global health; highlighting this generally overlooked but important area [48]. Kabaterine *et al.*[14] alluded to some of these complexities in the African context when they noted that restructuring several vertical programmes into one integrated MDA programme could “cause resentment among managers, in extreme cases leading to obstruction and other difficulties in managing the process”. Such human resources issues are embedded within different socio-cultural, economic and political processes that shape the governance and delivery of healthcare services [49].

Understanding national policy contexts is essential to adapting programmes from the global to national/local level. Civil service and health system reforms (i.e. sector-wide approaches, decentralisation and liberalisation) have been shown to present unique challenges; as noted by Cairncross *et al.* and Miri *et al.* in their analysis of the lengthy process of dracunculiasis eradication in Ghana and Nigeria [50, 51]. National policy contexts also foster differences in ministerial relationships between sectors that complicate inter-sectoral programmes, especially important for zoonotic NTDs such as rabies, cysticercosis, echinococcosis and Rhodesian sleeping sickness, as well as multi-sectoral programmes on water, hygiene and sanitation [52–54]. Complex fiscal relationships exist between different levels of government both vertically (from central to local) and horizontally (between ministries). Even where legislation exists, mechanisms to ensure inter-sectoral financial and operational responsibilities are often lacking – shown in a recent study on a dengue outbreak in Mexico [55]. Pharmaceutical liberalisation and the lack of state regulation can also contribute to inherent tensions between public health and other actors in the private health system – see Bardosh *et al.*’s analysis of the veterinary pharmaceutical market in sleeping sickness endemic areas of Uganda [56].

NTD governance arrangements involve many actors outside the realm of the state: international agencies, universities, the private sector, philanthropic foundations, international and national non-governmental organisations, community-based organisations and others [57]. Funding sources, institutional capacity and personal relationships are central to driving partnerships forward, where partners have different strengths and weakness, and power dynamics influence operational practices [58]. While non-state interventions implemented, for example, by NGOs, universities and the private sector can sometimes circumvent the hurdles of state bureaucracies and resource-constraints, long-term sustainability often demands state involvement or integration within the general health system; leaving the state behind, without capacity building, can create long-term problems in sustainability.

These various social contexts present multifaceted challenges to the scaling-up process. As discussed by a recent paper describing a USAID integrated MDA initiative in multiple African countries, scaling-up can involve: organising a central coordination mechanism, stakeholder engagement, situational analysis, action and work plans, funding gap analysis, disease mapping and defining monitoring and evaluations criteria [59]. While constraints are inevitable, their particulars are rarely subject to analytical scrutiny and the little attention given to monitoring and evaluation more generally risks limiting learning and adaptation [60]. The need for programmes to “look good” in order to attract donor-driven grant cycles have been noted for structuring what issues are researched and how, limiting opportunities to explore and discuss complex social and operational challenges [45].

Furthermore, the desire for results by donor agencies over short project cycles have been noted to push programmes to go to scale too quickly and lead to poor mobilisation, education and support for field staff. While a major challenge is enabling flexibility to country-contexts in NTD programmes that often have a global remit, arguably more challenging is adapting to socio-cultural, political, economic, infrastructural and environmental differences between and within districts. Studies show that despite trends towards decentralisation in local government in many endemic countries, feedback and responsiveness to local needs within the general health systems are limited; this is perpetuated by planning and managerial spheres being heavily centralised with little decision-making capacity at the district level – as shown by recent NTD studies in Tanzania, for example [61–63]. Transcending the classic “transplantation” problem in global health – where standardised approaches do not allow for context-specific strategies – requires thinking beyond the technology transfer model to engage more directly with local needs and the complexity of the scale-up process [64].

More than other areas, there is a dearth of research on policy processes for NTDs. Analysis of the interrelationships between global and national policy contexts, institutional and organisational norms and values, the devolution of power and information sharing, stakeholder incentives, and the potential bottlenecks in service delivery networks clearly require more attention. Such research can provide valuable information to help manage NTD programmes, build adaptability and learning into the scale-up process and exploit the strengths of different policy actors. With a variety of methodological and conceptual frameworks available, systematic health policy research could play important roles in informing NTD policy development and programme implementation [46, 65].

### II. Programmes and health systems

The second, albeit interrelated, priority area involves the interface between programmes and health systems. Functioning primary healthcare systems are recognised as essential to NTD control [66]. However neoliberal reforms in the late 1980s to “roll back the state” led to drastic reductions in health (and veterinary) sector expenditure in many developing countries [67]. At the district-level, programmes operate where human resources, information systems, essential drugs, basic infrastructure and supplies are often extremely limited. Weak state and patron-client relationships place local-level staff between the interests of the local and the national/global. The wider anthropological literature on international development shows that policy itself has a precarious influence on the operational practices of development projects – including NTD interventions – which are shaped more by organisational demands, norms and relationships at the local level [68, 69].

NTD programmes make use of health systems in three general ways: integration within health services (used for many leprosy programmes), structures completely outside (most mobile sleeping sickness screening services), and programmes organised by the central-level but carried out by district staff (many current integrated MDA programmes) [70]. There are both positive and negative impacts of such programmes on the general health system, ranging from effects on leadership and governance, health information systems, financing, infrastructure and supplies, workforce and service delivery [71]. One way to avoid negative impacts is to take account of resource-limitations and working norms *before* projects attempt to “piggy-back” on existing delivery networks; as shown by a scoping study on the feasibility of Human African Trypanosomiasis elimination in Zambia [72].

The few in-depth studies on the relationships between NTD programmes and health systems have shown different results. Baker *et al.*[73] reported a retrospective mixed methods study on two years of lymphatic filariasis integration in the Dominican Republic. This required adapting to national health sector reforms and decentralisation, including using community volunteers to account for staff shortages. The authors reported that integration substantially increased coverage of MDA (despite initial difficulties) and strengthened information systems and community engagement in the health system, partially because key stakeholders were well prepared for the integration process. Advocates of community-driven ivermectin treatment for onchocerciasis have argued that the approach strengthens primary healthcare by giving control of decision-making to communities, albeit providing the necessary resources and support requires multiple years of re-trainings [74]. In contrast, studies on leprosy programmes have shown the reluctance of control staff to take on extra responsibilities, the inadequacies of short trainings and variations between districts/regions based on the quality of the health system [75–77].

The few studies on MDA have also revealed significant challenges. Cavalier *et al.*[16] documented the disruption of access to general healthcare during integrated MDA in Mali and the lack of funds for institutional strengthening. A further ethnographic study in Tanzania commented on how district-level health staff considered handling multiple NTD interventions simultaneously as more of a burden than a cost-saving strategy [21]. Cairncross *et al.*[50] discussed 13 years of stagnation in dracunculiasis eradication in Ghana (due partially to the implementation of a sector-wide approach, government decentralisation and staff transfers) while Balen *et al.*[78] drew attention to more subtle social equity issues in China for schistosomiasis, where barriers to treatment were driven by the lack of medical insurance among the poor, despite a well planned and integrated programme.

Integrating programmes into the general health system is in many cases necessary and desirable. This requires vertical programme managers to navigate the political landscape of the health system, foster clear understandings of roles and responsibilities, and provide strategic training, resources, supervision and support. Local health systems remain frontline mediators between communities and neglected pathogens long after many donor-funded projects end; hence examining their needs, capacities and contexts should form an integral component of any comprehensive NTD programme.

### III. Community responses to interventions

A third key area for social inquiry to support NTD control involves community responses to interventions, particularly compliance and resistance. Among others, the work of the political scientists James Scott [79, 80] has shown how the intended beneficiaries of scientific interventions, including global health interventions, challenge dominant knowledge claims through local resistance, avoidance, noncompliance and strategic accommodation. Despite the best of intentions, technological interventions seldom involve the unproblematic application of scientific tools to local settings but invariably provoke acts of reinterpretation as they engage with local perceptions and experiences.

On the one end, the NTD literature shows that interventions are mediate by access barriers embedded within geography, delivery schedules and differences between population sub-groups. In a qualitative study, Mpanya *et al.*[81] noted the discrepancies between the schedule of a HAT mobile screening team and the movements of diamond miners and farmers, which significantly lowered the number of people willing to be tested. The need to tailor delivery strategies to different sub-populations is a ubiquitous finding of most social studies; as shown in the influence of gender on MDA [82], compliance of pastoralists with canine vaccination for rabies [62] and schistosomiasis control among fisherfolk [19]. Numerous other access barriers have also been noted. Omedo *et al.*[83] in Kenya argued that better coverage for schistosomiasis control would be achieved if MDA were carried out during the harvest period to correspond with food availability to reduce side-effects. Coverage of ivermectin treatment has been low due to discontinuities between the working hours of health personnel and farming schedules - a partial impetus for allowing community volunteers to determine the period of treatment [74]. Furthermore, the success of case detection strategies and surveillance, for example in visceral leishmaniasis control in South Asia [84], can depend substantially on whether communities prefer public or private health systems and how projects engage with different providers. Clearly, conducting such studies prior to a biomedical research or control programme can avoid many costly (and unnecessary) operational blunders.

However barriers to community compliance go well beyond these more easily remedied operational issues and a growing body of literature on “local resistance” to global health interventions has emerged [85–87]. Resistance is often deeply rooted in local frames of reference, tied to social networks and related to wider socio-political issues. Local understandings of disease, relationships to government officials and the state, and past experiences with development projects play important mediating roles. For example, local perceptions about drug toxicity, lack of confidentiality during screening procedures and local beliefs about the costs of treatment contributed to resistance to HAT screening in the DRC [81]. Hastings [87] described how riots occurred after an MDA programme in Tanzania due to fears that it was a covert sterilization campaign, which resonated with local anxieties questioning the intentions behind western-driven development projects. In fact, local resistance to MDA has been reported in numerous studies in the last few years [17–21, 88–95]. A review of research for lymphatic filariasis control identified: local fears over treatment and side-effects, individual characteristics (including mobility patterns), knowledge and awareness, prior experiences with MDA and the training, motivations and characteristics of distributors [90]. Observing the poor compliance rates for MDA in Ugandan schools, Muhumuza *et al.*[94] proposed increasing health education, teacher motivation and providing snacks. Others have questioned the effectiveness of using teachers and community volunteers where notions of medical expertise are exclusively tied to health professionals [87, 95]. A more complicated picture was illustrated by ethnographic fieldwork done by Parker and Allen [17–20] who remarked on the need to link MDA drug distribution to more holistic control approaches to legitimise it to reluctant community members – confused about why they should swallow tablets when disease drivers remain unaddressed.

Understanding livelihood patterns, migration, health seeking behaviour, local knowledge and others social processes at play among the “bottom billion” can help tailor programmes to enhance community acceptability. A major issue is the level of trust between recipient communities and field staff and delivery networks, and the degree to which interventions are tailored to local concerns, perspectives and needs. Giving greater scope to local voices would address many, but perhaps not all, cases of “active resistance”. There is a need to abandon the simplistic, but safer, interpretation that noncompliance is primarily about “lack of awareness” and reflect more on the nature of the programme strategies used and their wider socio-political context – what has been called the political epidemiology of disease control [96].

### IV. Educating publics, changing behaviours

While risk behaviours have been widely emphasised by social science studies on different NTDs [97, 98] little research has directly and systematically explored health education, despite its importance for addressing non-compliance. For example, the studies referred to above on MDA are unanimous that certain information is fundamental to accompany MDA but have not been readily incorporated into campaigns: Why do some experience side-effects but others not? Why is praziquantel given by height and not weight? Why do people without symptoms have to take drugs? Why is the programme not addressing water and sanitation? Education strategies play a double role. They not only promote greater community compliance by making people aware of the value of participating in a given programme but they can also be used to promote fundamental behaviour changes with long-term implications.

There is a large literature on the complexities of devising and tailoring education and public engagement strategies for health issues in developing country contexts; the literature emphasises that messages should not only be culturally acceptable but “culturally compelling” [99]. In a revealing study on malaria nets in Africa, Panter-Brick *et al.*[100] showed the need to reinforce simple messages over time using different mediums, gain community support and focus on positive outcomes while also addressing constraints to people’s agency. Similarly, based on his experience with guinea worm, Brieger [101] emphasised moving beyond didactic methods to include skills development, knowledge acquisition and active community involvement.

Few studies explore how existing national programmes educate their publics for NTDs and how education campaigns can move beyond small-scale, donor-funded research projects. An ethnographic study in Cambodia on the national dengue control program delivery at schools, health centres and villages is a rare and insightful example. Under-funded, irregularly implemented and seldom evaluated, Khun and Manderson [102] reported on various operational shortcomings, including little training or incentives for health staff and teachers and confusing messages that did not engage with local constraints to behavioural change. The authors recommended schools engage in community-based vector control outreach activities to move beyond an otherwise passive education model. The work of Parker and Allen [17–20] is also informative. In Uganda and Tanzania, they found that adults were increasingly rejecting free treatment for schistosomiasis, lymphatic filariasis and soil-transmitted helminths. Part of this involved health education used by volunteers, school teachers and government officials that did not engage with real and imagined fears of side effects based on local aetiological concepts and suspicions about the motivation of the programme. Similarly, Hastings [87] revealed how health education accompanying an MDA programme in Tanzania did not provide sufficient time for dissemination to parents and local leaders. Influenced by time and budget restrictions, Burke [103] also noted the “top-down” planning process used in Togo during an integrated MDA health education strategy which did not engage with gender dimensions, local understandings of disease, drug side-effects and the rationale behind MDA, although community volunteers did adapt guidelines in practice.

Given emerging critiques of non-compliance to NTD interventions, especially MDA, there is an urgent need to explore how national NTD programmes are engaging with community perceptions in health education and how future educational strategies can address local concerns. Although most of the NTD literature shows positive increases in knowledge through health education [104–106], few studies explore the opportunities and challenges involved in how education can be used to actually change behaviour – see El-Katsha and Watt’s superb study on schistosomiasis in Egypt (with a focus on gender differences) for an example [107]. There are deeply imbedded barriers to behaviour changes that need to be considered and incorporated into educational strategies [108]. For example, the tensions between the public and private goods aspect of vector control has been noted for why people, otherwise knowledgeable about prevention practices, do not practice them [109, 110]. Important differences between (sub-) populations may require different strategies, based on gender, age, wealth and livelihoods [111, 112]. These differences maintain power dynamics at the community-level that need to be understood. Broader disjunctions between “knowing” and “doing” are also structured by poverty, livelihood patterns and locally embedded socio-cultural processes, which need to be more thoroughly accounted for during the planning of interventions. Finally, there is also clearly a need to consider the fact that behaviour change, even within a well-designed programme, may be unlikely to occur in politically and economically-marginalised communities, at least for certain types of embedded behaviours. Broader changes in economy, politics and social organization may be needed as prerequisites for effective, long-term disease control.

### V. Fostering participation, tailoring programmes

The last priority area, intersecting with the others, relates to community participation, which is often polarised between dismissal and romanticisation. Engaging with communities occurs in different ways depending on envisioned goals and outcomes but is arguably the most essential aspect of addressing local resistance and fostering behaviour change. Rifkin [113] divided community involvement into a spectrum from passive compliance oriented around a “target framework” to active involvement in priority setting and planning focused on “empowerment processes” where planners and implementers take on roles as facilitators. The NTD literature shows this full spectrum of participatory strategies; for example, mobilising households in triatomine surveillance [114], controlling schistosomiasis through changes in water contact [115] and irrigation system cleaning [116], volunteer networks for guinea worm eradication [117], and grassroots task forces for dengue control [118]. This literature shows us that without a sound understanding of community dynamics guiding intervention planning there is a risk that planners and implementers pursue reductionist strategies.

“Communities” rarely adhere to geographical locations, which are composed of a diversity of sub-groups with differences in needs, capacities and constraints. This may present challenges for approaches that seek the broad involvement of the community; for example, community-led total sanitation (CLTS) – a participatory sanitation programme where people are encouraged to build their own locally-sourced pit latrines and recently promoted by NTD advocates – may face challenges in addressing entrenched gender dynamics in Africa [119]. While compelling, the wider development literature points to numerous potential shortcomings when participatory interventions are scaled-up from small-scale successes: the tendency for marginalized groups to be excluded, facilitators to dominant the process, lack of awareness of conflicting interests at the community-level, pressures for quick results and the use of self-interested intermediates [120, 121]. Perhaps more problematic is the notion that community participation involves communities “doing it themselves” which overlooks the fact that such interventions essential create new social networks dependent on the implementing agency. The failure of many community-driven tsetse trap projects popular in the 1990s and 2000s is one example [110]. People were willing, to varying degree, to contribute money, time and effort but activities ceased in the absence of inputs and state/NGO/academic coordination [122, 123]. While some have argued that technical experts hampered success by never truly devolving ownership [123], poor farmers are also reluctant to invest scarce resource and time into a public good, especially once vector populations decline.

Past experiences with tsetse trap projects, and many other participatory NTD efforts, questions the notion that sustainability equates to the cessation of support from the outside. Clearly, economic and political realities reduce the resources, time and capacity of “the bottom billion” to participate in disease control and seek medical treatment in dynamic ways. Although this questions the idealistic notion that community members can identify, prioritize and resolve their own health problems [41], it also reinforces the need for community-led initiatives to help build societal resilience. Rather, sustainability should be evaluated by how projects facilitate continued linkages between communities and their local health system, and other actors. One way to do this is to integrate acceptable and efficacious strategies that resonate broadly with the lives of poor people through addressing sanitation, water, primary healthcare, housing and environmental management instead of narrowly focussing on one specific pathogen – NTDs are only one problem among many facing the lives of the poor.

Furthermore many successful programmes are built not on mass community mobilisation but on enrolling the support of key actors at the community-level. Much debate has ensured about what type of inputs should be provided and the level of devolved priority setting and planning. The most well documented example for an NTD involves community-directed ivermectin treatment for onchocerciasis in Africa, emerged from a lengthy process of operational trial, error, learning and adaptation since the 1980s [74]. Volunteers are selected by the community, trained and given ownership over determining the period and mode of annual treatment. Although financial incentives are rarely provided, social status (recognition, self-fulfilment and political influence) and meals, books and labour from recipients act as important non-monetary incentives [124, 125]. The motivation and support offered to volunteers clearly impacts coverage. Emukah *et al*. [126] reported on the high drop-out rate of distributors in Nigeria (at least 35%) related to a lack of incentives and supervision, long travel distances, other duties and poor supply of ivermectin. Other studies have commented on the fact that higher coverage is maintained by volunteers living closer to the communities they serve and being responsible for fewer households [124, 127]. The involvement of women in patriarchal societies is also a significant operational issue [128] while volunteers can be overburdened and/or dissuaded by other community-based activities (such as polio vaccination) where money is provided [129]. Variations between countries based on the strength of the health system have also been noted [124].

There are a number of areas for implementation research to explore innovative and practical ways for community participation to enhance NTD efforts. The first is how existing community participatory strategies, for example volunteer networks in ivermectin, can strengthen primary healthcare which, despite much discussion, has not been the subject of in-depth social studies. Arguably, greater resources and support are required to be channelled to the community-level for this to be realised. Second, there are few examples of projects that seek to engage different actors in multi-stakeholder strategies where different concerns and interests are incorporated into the planning process. For example, a dynamic 10 year action-research process for the control of cystic echinococcosis in Nepal moved from biomedical strategies to focussing on garbage collection policy and urban renewal through a stakeholder engagement process [130]. Other multifaceted examples include the control of dengue in Latin America [131] as well as a 15 year partnership between mobile pastoralists, government authorities and the Swiss Tropical and Public Health Institute in the Sahel [132]. Studies should more systematically evaluate their feasibility and benefits as a way to strengthen relationships between communities, social service providers and donor-projects. Third, there is a widespread lack of acknowledgement of the types of participatory interventions that are most effective in NTD control and how they can be scale-up efficiently. To address this, social science research is needed to devise new methodologies to measure behaviours, change processes and social impact in order to provide the evidence-base for incorporating community participation into mainstream NTD programmes, while also avoiding the tendency for participation to become a technocratic, tick-the-box exercises [133]. Fourth, there remains much scope to explore the shortcomings of how contemporary interventions are engaging communities, where social scientists can address barriers and propose new strategies, preferably as part of a long-term engagement strategy in collaboration with biomedical and public health experts [134].

## Conclusions

This paper has discussed a range of social dynamics involved in moving global health interventions from management boardrooms in Geneva, London, Washington, Nairobi, Bangkok and elsewhere to the many tens-of-thousands of villages and slums where Neglected Tropical Diseases persist among marginalised populations. Given recent targets set for 2020, the coming years will see many programmes expanded and scaled-up. However as this paper has shown, there are fundamental and multifaceted challenges facing planners, implementers and communities in sustainably controlling NTDs. Pre-empting and addressing these as part of a critically engaged social science research agenda is an essential aspect of good science, sound management and global ethics. *The Global Report for Research on Infectious Diseases of Poverty* (2012) outlined a compelling rationale for addressing gaps in implementation [30, 31]. However such conceptually sophisticated plans themselves have barriers to uptake and diffusion related to funding priorities, expertise, the level of collaborative work between social and biomedical scientists and the ability for research to influence policy and practice [32–40].

Apart from discussing specific research areas where the social sciences can support interventions (see below for a summary), this paper also makes a number of implicit and interrelated arguments; these are supported not only by the existing NTD social science literature (including the conclusions of many of the above cited research papers) but also the wider anthropological literature on global health [85, 86, 135–137]. The first relates to the fact that, while insightful social research has certainly been conducted on many NTDs, such research continues to be relatively marginal compared to biomedically-orientated priorities and perspectives. This general lack of social inquiry on control programmes serves as a proxy for the nature of information and knowledge flows in the NTD global network. Good social inquiry engages in multidimensional ways with the complexities of a given real world problem as embedded within its socio-cultural, economic, political and environmental contexts. It involves spending time talking with people, asking difficult questions, challenging common assumptions, incorporating and making sense of divergent views and generating novel interpretations based on “thick descriptions”. It involves understanding the local in context and as process. Such inquiry helps to transform amorphous and compliant “populations” into active “publics” who have agency, opinions and contexts that shape how they view and engage with outside plans and strategies [138]; in short, it offers the “bottom billion” a chance to become involved in global health governance and assist in setting priorities and tailoring programmes.

**Priority areas for social research to support NTD control**

1. **Policy Processes**

- How global/national policy contexts influence NTD interventions
- What institutional and organisational norms and values mediate programme operations
- The devolution of power and information sharing between partners
- Social dynamics of data generation and use
- Stakeholder incentives and drivers
- Assumptions and narratives around scaling-up

2. **The Interface Between Programmes and Health Systems**

- Existing resource-limitations and working norms of health systems
- Needs, capabilities and context of the primary health system and implementers
- Ways to synchronise programme goals with primary health system strengthening
- Effects of interventions on general health services

3. **Community Responses to Interventions**

- Access barriers, such as how geography, sub-populations and delivery schedules influence delivery
- Incentives for implementers to deliver interventions
- How local understandings of disease, livelihood patterns and other social processes influence coverage and adoption of health technologies

4. **Education and Behaviour Change**

- The social, cultural, political and economic barriers to behavioural changes
- Needs of differences sub-populations for tailored strategies
- How existing national programmes educate their publics
- How education strategies can move beyond small-scale, donor-funded projects to be scaled-up effectively

5. **Community Involvement and Participation**

- Social diversity at the community level and how it affects participatory processes
- Scope to link NTD control with wider issues in sanitation, water, primary healthcare, housing and environmental management
- Feasibility and processes involved in multi-stakeholder engagement strategies
- The shortcomings of how contemporary interventions are conceptualising and engaging communities
- The trade-offs between short-term and long-term community engagement

However in global health more generally, class and cultural barriers between global and local actors reinforce an unequal power dynamic (itself a representation of global poverty and inequality) whereby the flow of knowledge and information goes downwards from the global to the local but little evaporates upwards, at least outside epidemiological data, coverage numbers and risk factor studies [135]. If programmes are to be truly “pro-poor” then this power dynamic should be acknowledged and addressed more proactively by incorporating the voices, opinions, experiences and capacities of the “bottom billion” more explicitly into policies and programmes, and fostering more people-centric interventions. Otherwise global NTD policy narratives can begin to replicate inequality – or even colonial discourses of the “other” – by objectifying the very people they seek to assist. As noted by Vlassoff [40] more than two decades ago, tropical disease interventions can all too easily over-emphasise a narrow focus on pathogens and technologies, neglecting social perspectives.

If local realities are to be better linked to technical solutions then a re-conceptualisation among biomedical scientists, donors and ministerial officials that the “soft sciences” only provide “anecdotal” evidence, is only useful in uncovering “exotic cultural practices” and consists of “nothing but” a standardised questionnaire is needed. Despite decades of (continuing) research funding (i.e. by TDR) aimed at fostering multidisciplinary teams and collaboration between social and biomedical scientists, it is clear that the actual implementation of many contemporary NTD interventions underutilise the potential of social inquiry. Such perspectives extend far beyond the standardised “knowledge”, “attitudes” and “practices” questionnaire that the social sciences are often reduced to in global health [139].

In many ways, qualitative and mixed methods research offers one of the most cost-effective research strategies for improving programme operations, if findings are readily incorporated into intervention plans and strategies. There are, of course, numerous challenges to seeing this type of data generated, read, considered and used in global health, including for NTDs. As one anonymous reviewer pointed out, many of the central arguments of this paper have simply reiterated points discussed since the 1970s about the relationships between the social and biomedical sciences [40]. Traditional disciplinary differences are maintained by different systems of reward and expectations, further reinforced by funding bodies and grant cycles and the socio-political nature of development institutions and organizations. Despite the rhetoric of multi-disciplinarity, it is clear that entrenched silos remain. This requires donor agencies and national governments to conceptualise new ways of conducting research and programmes to move social sciences from an afterthought to a core component of mainstream donor and national activities.

Given the large geographical areas of many programmes, social scientists should also be challenged to engage more with rapid assessment techniques to support collaborative team efforts with biomedical scientists [140]. Concerted efforts to build social science capacity in ministries and universities in endemic countries should also continue to be pursued as well as efforts to foster inter-departmental platforms. Greater reflection on how to design studies to generate an evidence-base to stimulate new people-centric intervention strategies and convince programme managers and policymakers of their practical benefits are essential, blurring the line between research and action. Reflection on the acknowledged perils of “rapid ethnography” need to be negotiated as research straddles, perhaps uneasily, the segregation of being both *in* and *out* of the public health establishment [141]. While critical perspectives offer important insights, as Kleinman [142] commented in *The Lancet* there is a tendency for social scientists, driven by structural systems of reward within their own epistemic communities, to valorise attacking biomedicine and public health without specifically attempting to improve it. How can social scientists remain critical but yet focused on building on, improving and working with biomedical and development actors?

My final argument relates to the influence of greater social inquiry on NTD interventions. Giving greater scope to social perspectives may drive a shift where new ways of conceptualised and implementing programmes takes place. At present, much attention on NTDs has focused on a narrative offering poverty reduction through low-cost interventions, particularly around MDA. However there are inherent trade-offs and tensions between these goals. Promises of “rapid impact packages” are likely to focus on narrow biomedical approaches that engage little with the social determinants of health and illness [10]. The value of such vertical interventions has been much debated since the Alma Ata Declaration (1978), often in highly polarised terms [137]. These tend to frame debates from an *either/or* perspective. *Either* programmes efficiently reduce the health burden of select illnesses through “narrow biomedical” approaches (or “military operations”) *or* they improve health systems by promoting governance reforms, addressing of social determinants and citizen engagement. In the end, vertical NTD approaches offer more attractive, a-political and relatively simplified cause-effect narratives more effective in mobilise resources and amenable to conventional data reporting structures. They have also, as Molyneux and Malecela [3] correctly point out, achieved many noteworthy public health successes.

Rather the question is: how can these “cost-effective” strategies also engage in the larger challenge of addressing the under-lying drivers to neglected diseases and help build long-term societal resilience? Many of the research papers reviewed here concluded that more could have been done to link existing interventions with these broader social concerns. However, it is one thing to criticise contemporary efforts and quite another to actively engage with the conundrum of linking public health to wider developmental challenges and concerns. Focused on the Avian Influenza response in Asia, Scoones [86] has shown, in relation to his critique of the One World, One Health movement, that moving from an expert-driven, top-down approach to focus on long-term societal change is difficult to imagine, and yet still more difficult to put into practice. Global health actors navigate a complex socio-political landscape that requires framing evidence, mobilising resources and support, obtaining results and presenting data back to funding bodies to sustain activities. Broader goals, such as “improving daily living conditions” and “tackling the inequitable distribution of wealth, resources and power at the global, national and local scale” (as recommended by the WHO’s Commission on the Social Determinants [26]) present somewhat of a challenge to the expectations, norms and capabilities that regulate these networks. While this may seem an obvious point, the realisation that our ever-expanding scientific knowledge-base has far outpaced our ability to effectively deal with the social determinants of disease should challenge funders, practitioners and researchers to think in more creative ways [143]. Otherwise poor populations may find that while one NTD has been eliminated, another one has simply moved in to take its place.

Beyond simplistic dichotomisations, there is much scope for disease-specific interventions to engage broadly with poverty and marginalisation, especially if we conceptualise NTD control not from the short project cycle but, as Zhang *et al*. [144] noted, based on a long-term commitment. This requires a shift in donor funding that emphasises engaging in larger societal and developmental processes. A number of commentaries, mostly from Latin America, have emphasised ways that NTD control can create synergies and collaborative programmes with other sectors to ddress the social determinants of health, including education, housing, water and sanitation, public works, agriculture, the private sector and economic development [6]. Other less expansive recommendations emphasise coupling programmes with the delivery of other health technologies, such as mosquito net distribution, nutrition programmes and vitamin A distribution [145–148]. Other opportunities exist to link the control of zoonotic diseases of poverty to agricultural development and the provision of animal health services at the community-level, as shown by the Stamp out Sleeping Sickness programme in Uganda [56].

This paper agrees with other recent critiques of global health that have emphasised the tendency for local realities to be put into the margins during planning, implementing and monitoring and evaluating interventions. Adams *et al.*[149] called for a movement in “slow research” to parallel the “eat locally” food movement in order to critically inspect the processes involved and their multiple associations. Also using metaphor, Panter-Brick, Eggerman and Tomlinson [150] outlined what they called the need to master the four “deadly sins” of global health through a “change of heart”: the coveting of silo gains, lusting after technological solutions, boasting of small successes and leaving broad promises unfulfilled. Within this wider context and critique, NTD actors have an opportunity to push boundaries and help move infectious disease control towards more resilient and sustainable pathways as envisioned in a One World, One Health agenda, not to mention influencing the post-MDG developmental agenda in dynamic, new ways [5]. Greater attention to social inquiry can help foster new ways of engagement and orientation. For this to happen, collaborative and transdisciplinary research needs to be accompanied by changes in governance structures (including funding streams) towards more flexible models that account for diversity of perspectives, seek to understand local complexities and aim to promote adaptation, reflexivity and learning over time [151, 152]. Short project cycles that dissuade critical analysis, local capacity building and long-term engagement are problematic. In conclusion, the increased attention and funding for NTDs has the potential to alleviate some of the devastating effects of major infectious disease on the world’s poor. With bold new targets established for 2020, however, it is high time that local realities and social science perspectives are put at the forefront of intervention planning.

## Electronic supplementary material

Additional file 1:Multilingual abstracts in the six official working languages of the United Nations.(PDF 235 KB)
