# Supplementary material for: Global aspirations, local realities: the role of social science research in controlling neglected tropical diseases
Source: Infect Dis Poverty. 2014 Oct 1;3:35. doi: 10.1186/2049-9957-3-35 (PMC4197218; doi:10.1186/2049-9957-3-35)
Supplement: Supplementary file 1 — Additional file 1:Multilingual abstracts in the six official working languages of the United Nations.(PDF 235 KB) [file 40249_2014_78_MOESM1_ESM.pdf]

Translation of the abstract into the six official working languages of the United Nations

تطلعات عالمية، وقائع محلية: دور البحث العلمي في التحكم في الأمراض الإستوائية المُهملة

كيفين باردوش

**ملخص**

إن الأمراض الإستوائية المُهملة (NTD) هي أسباب ومظاهر على حد سواء للفقر وعدم العدالة الاجتماعية في الدول النامية. وقد أدت الدعوات المتزايدة منذ منتصف عام 2000 إلى وضع منظمة الصحة العالمية لأهداف طموحة وجديدة للتحكم هذه الأمراض وإنهائها بحلول عام 2020. بينما تمثل تلك التطلعات العالمية سياسة زخم هامة، فإن تحريك الأدوات والاستراتيجيات الحالية الخاصة بالتحكم إلى السياقات محلية فقيرة الموارد يقدم تحديات كبيرة. وقد ركزت عدد من المنشورات الحديثة على الحالة "المُهملة" نسبياً لأبحاث العلوم الاجتماعية المطبقة على الأمراض الإستوائية المُهملة (NTD). في ضوء أهداف 2020، تقوم هذه الورقة بسبر أغوارالعلم الاجتماعي/ أدبيات الأمراض الإستوائية المُهملة (NTD) والكشف عن بعض الطرق التي يمكن من خلالها للبحث الاجتماعي أن يساعد في دعم التدخلات الفعالة والمستدامة. ومن أجل هذه الغاية، تقدم الورقة خمسة مجالات ذات أولوية، وتضم العمليات السياسية وقدرة الأنظمة الصحية والالتزام ومقاومة التدخلات والتعليم والتغيير السلوكي والمشاركة المجتمعية. وسوف يتم تحليل كل من الأدبيات السابقة ومناقشة المجالات المستقبلية للبحث بصورة موجزة. يُظهر تخطيط البحث الاجتماعي الحالي بوضوح القيمة متعددة الأوجه لإدماج المنظورات الأنثروبولوجية والاجتماعية بشدة في البرامج الخاصة بالأمراض الإستوائية المُهملة (NTD). ولكن يبدو أن الجهود المعاصرة فشلت في استخدام هذه الإمكانية، مما يعكس سيطرة تدفق المعلومات من الأعلى للأسفل والتوجهات التقنوقراطية في الصحة العالمية. ومن أجل مقاومة هذا التوجه، يحتاج البحث الاجتماعي إلأى أن يكون أكثر من مجرد أمر ثانوي؛ وأن يتم إدماج البحث الاجتماعي في داخل التخطيط وسوف تساعد مراقبة العملية وتقييمها على ضمان أن يتم بناء المرونة والتكيف في الوقائع المحلية في داخل التدخلات. كما يمكن أن يساعد المزيد من التركيز على المنظورات العلم الاجتماعي على ربط التحكم في الأمراض الإستوائية المُهملة (NTD) بمحددات اجتماعية أوسع نطاقاً للصحة، مع إعطاء أهمية خاصة لعدم المساواة الاجتماعية والاقتصادية الكبرى التي ترتكز على الانتقال في الدول المتوطنة.

Translated from English version into Arabic by Reham Hussien, through

TRANSLATORS
WITHOUT BORDERS

**全球愿景和地区现实：社会学研究在被忽视热带病控制中的作用**

Kevin Bardosh

**摘要：**

被忽视热带病是发展中国家贫困和社会不均衡的因素和反映。自21世纪前十年中期开始，越来越多的呼吁促使 WHO 制定了雄心勃勃的2020年控制和消除目标。尽管这些全球目标反映了重要的政策推动，但是如何将已有的控制措施和策略应用于不同的、资源有限的当地环境中仍然面临诸多挑战。近来发表的众多文章均表明社会学研究在被忽视热带病中应用的不足。鉴于2020目标，本文通过分析社会学和被忽视热带病方面的文献，阐明如何应用社会学方法来帮助实施有效的和可持续的干预。本文提炼出五个优先领域，即政策制定、卫生系统能力、干预的依从性和抵抗、教育和行为改变及社区参与；分析了以上各个领域的相关文献并且简要讨论了未来的研究方向。已有的社会学研究清楚地表明将人类学和社会学整入被忽视热带病规划中的多方面价值。当然，目前这种价值还没有得到充分发挥，表明全球卫生中仍然以自上而下的信息流动和官僚式技术方法为主。为改变这一趋势，社会学研究就不能仅仅是事后才考虑的事，而应该在计划、监测和评价过程中就要考虑，以确保在当地环境下干预措施具有弹性和适应性。对社会学的重视能够

促使被忽视热带病和更广层面的卫生相关社会决定因素之间的联系，鉴于社会和经济不均衡导致流行国家传播继续，这点就尤为重要。

Translated from English version into Chinese by Qian Men-bao, through

**Aspirations mondiales, réalités locales : le rôle de la recherche en sciences humaines dans la lutte contre les maladies tropicales négligées**

Kevin Bardosh

**Résumé**

Les maladies tropicales négligées (MTN) sont des facteurs de la pauvreté et d'inégalité sociale dans les pays en voie développement et en sont aussi la manifestation. Des efforts accrus de sensibilisation depuis le milieu des années 2000 ont donné lieu à d'ambitieux nouveaux objectifs en matière de lutte et d'éradication établis pour 2020 par l'Organisation mondiale de la Santé. Bien que ces aspirations mondiales représente un élan politique important, faire passer les outils et stratégies de lutte à différents contextes locaux offrant peu de ressources présente plusieurs défis. Un certain nombre de publications récentes ont mis l'accent sur le statut relativement "négligé" de la recherche en sciences humaines appliquées sur les MTN. À la lumière des objectifs pour 2020, cet article explore la littérature sur les sciences sociales/MTN et dévoile certaines façons dont une enquête sociale peut appuyer des interventions efficaces et durables. À cette fin, l'article présente cinq domaines prioritaires, notamment les processus d'élaboration des politiques, la capacité des systèmes de santé, le respect interventions et la réticence à celle-ci, l'éducation et les changements comportementaux et la participation communautaire. Pour chaque domaine, une littérature récente est analysée et les futurs domaines de recherche sont brièvement abordés. L'ensemble de la recherche sociale existante démontre clairement la valeur d'une perspective anthropologique et sociologique fermement intégrée dans les programmes liés aux MTN. Cependant, les efforts contemporains semblent sous-utiliser ce potentiel, reflétant la dominance d'une information descendante et d'approches technocratiques en matière de santé mondiale. Pour inverser cette tendance, la recherche sociale doit aller plus loin que la réflexion; intégrer une enquête sociale au processus de planification, de surveillance et d'évaluation aidera à s'assurer que flexibilité et adaptabilité aux réalités locales soient intégrées aux interventions. Une plus grande importance accordée à l'angle des sciences sociales peut également aider à faire un lien entre la lutte contre les MTN et les déterminants sociaux plus larges de la santé, ce qui est tout particulièrement important, étant donné les inégalités socioéconomiques majeures qui continuent de favoriser la transmission dans les pays endémiques.

Translated from English version into French by BM1983, through

**Глобальные цели, местные реалии: роль социологических исследований в контроле забытых тропических болезней**

Кевин Бардош

**Тезисы**

Забытые тропические болезни (ЗТБ) являются как причиной, так и следствием бедности и социального неравенства в развивающихся странах. Под влиянием общественности, которая с середины 2000-х годов стала занимать все более активную позицию по отношению к этой проблеме, ВОЗ разработала обширные новые цели по контролю и искоренению этих болезней к 2020 году. Хотя эти глобальные усилия и имеют большую значимость, использование существующих средств контроля в совершенно ином местном контексте при острой нехватке ресурсов представляет собой особую сложность. Относительно «забытая» роль прикладных социологических исследований в области ЗТБ подчеркивается в нескольких недавних публикациях. С учетом поставленных на 2020 год задач в данной статье анализируются публикации, касающиеся социологических исследований / ЗТБ, а также объясняется, каким образом они могут помочь в проведении эффективных и дающих устойчивые результаты мероприятий по борьбе с данными болезнями. С этой целью в работе рассматриваются пять главных тем: формирование государственной политики, возможности здравоохранительной системы, сопротивление и содействие мероприятиям по борьбе с болезнями, просвещение и изменение образа жизни, а также задействование местного сообщества. Далее в статье анализируются последние публикации по каждой из этих тем и кратко обсуждаются новые направления для исследований. Существующие социологические работы ярко демонстрируют, насколько важно учитывать антропологические и социальные аспекты при определении стратегий по борьбе с ЗТБ. Несмотря на это, их потенциал не используется в полную меру в существующих программах: при их разработке предпочтение отдается передаче информации «сверху-вниз» и технократическому подходу к проблемам всемирного здравоохранения. Для борьбы с этой тенденцией социологическим исследованиям необходимо уделять особое внимание и учитывать их при планировании, мониторинге и оценке ситуации, что в значительной степени будет способствовать большей гибкости и приспособленности различных мероприятий к местным реалиям. Социологический подход к проблеме может помочь контролю ЗТБ охватить более обширные социальные факторы здоровья, что особенно важно в эндемических странах, где серьезные социально-экономические неравенства продолжают способствовать распространению болезней.

Translated from English version into Russian by Irina_Ka, through

TRANSLATORS
WITHOUT BORDERS

**Aspiraciones Globales, Realidades Locales: El papel de la Investigación en Ciencias Sociales en el control de las Enfermedades Tropicales Desatendidas (ETD)**

Kevin Bardosh

**Extracto**

Las Enfermedades Tropicales Desatendidas son tanto creadoras como manifestaciones de la pobreza y la desigualdad social en los países en vías de desarrollo. Los esfuerzos de apoyo, incrementados desde mediados del año 2000,  han dado lugar a nuevos objetivos ambiciosos de control y eliminación, los cuales han sido fijados para el año 2020 por la Organización Mundial de la Salud. Si bien estas aspiraciones globales representan, dentro de la política, un impulso significativo, las herramientas y estrategias de control existentes en los diferentes contextos locales con escasos recursos, presentan numerosos retos. Varias publicaciones recientes han puesto de manifiesto la situación relativamente "desatendida" de investigación en ciencias sociales aplicadas a las ETDs.  A la luz de los objetivos para 2020, este artículo explora las ciencias sociales/literatura ETD y expone algunas de las formas en que la investigación social puede ayudar a apoyar intervenciones eficaces y sostenibles. Con este fin, el artículo presenta cinco áreas prioritarias, la inclusión en los procesos políticos, la capacidad de los sistemas de salud, la conformidad y la resistencia a las intervenciones, la educación y el cambio en el comportamiento y la participación comunitaria.  Para cada uno se analiza la literatura reciente y se discuten brevemente futuras áreas de investigación. El panorama de la investigación social existente muestra claramente el valor multifacético de tener perspectivas antropológicas y sociológicas firmemente integradas en los programas de ETD. No obstante los esfuerzos actuales, este potencial parece haberse infrautilizado, reflejando el dominio de la información de arriba hacia abajo de los flujos y los enfoques tecnocráticos en la salud mundial. Para contrarrestar esta tendencia, la investigación social tiene que ser más que una idea de último momento; la integración de la investigación social en la planificación, el seguimiento y la evaluación del proceso ayudará a asegurar que la flexibilidad y la capacidad de adaptación a las realidades locales sean incorporadas en las intervenciones. Un mayor énfasis en las perspectivas de las ciencias sociales también puede ayudar a enlazar el control  de las ETDs con determinantes sociales de salud más amplios, siendo especialmente importante dadas las grandes desigualdades sociales y económicas que siguen sustentando la transmisión en los países endémicos.

Translated from English version into Spanish by patriciacassoni, through

TRANSLATORS
WITHOUT BORDERS
